# Modulated expression of the HIV-1 2LTR zinc finger efficiently interferes with the HIV integration process

**DOI:** 10.1042/BSR20181109

**Published:** 2018-09-07

**Authors:** Sutpirat Moonmuang, Somphot Saoin, Koollawat Chupradit, Supachai Sakkhachornphop, Nipan Israsena, Ruttachuk Rungsiwiwut, Chatchai Tayapiwatana

**Affiliations:** 1 Center of Biomolecular Therapy and Diagnostic, Faculty of Associated Medical Sciences, Chiang Mai University, Chiang Mai 50200, Thailand; 2 Division of Clinical Immunology, Department of Medical Technology, Faculty of Associated Medical Sciences, Chiang Mai University, Chiang Mai 50200, Thailand; 3 Research Institute for Health Sciences, Chiang Mai University, Chiang Mai 50200, Thailand; 4 Stem Cell and Cell Therapy Research Unit, Faculty of Medicine, Chulalongkorn University, Bangkok 10330, Thailand; 5 Department of Pharmacology, Faculty of Medicine, Chulalongkorn University, Bangkok 10330, Thailand; 6 Department of Anatomy, Faculty of Medicine, Srinakharinwirot University, Bangkok 10900, Thailand

**Keywords:** Designed zinc-finger protein, Gene therapy, HIV-1, Pluripotent stem cells, Tet-On system

## Abstract

Lentiviral vectors have emerged as the most efficient system to stably transfer and insert genes into cells. By adding a tetracycline (Tet)-inducible promoter, transgene expression delivered by a lentiviral vector can be expressed whenever needed and halted when necessary. Here we have constructed a doxycycline (Dox)-inducible lentiviral vector which efficiently introduces a designed zinc finger protein, 2-long terminal repeat zinc-finger protein (2LTRZFP), into hematopoietic cell lines and evaluated its expression in pluripotent stem cells. As a result this lentiviral inducible system can regulate 2LTRZFP expression in the SupT1 T-cell line and in pluripotent stem cells. Using this vector, no basal expression was detected in the T-cell line and its induction was achieved with low Dox concentrations. Remarkably, the intracellular regulatory expression of 2LTRZFP significantly inhibited HIV-1 integration and replication in HIV-inoculated SupT1 cells. This approach could provide a potential tool for gene therapy applications, which efficiently control and reduce the side effect of therapeutic genes expression.

## Introduction

 HIV infection continues to be a major global health problem. Highly active antiretroviral therapy (HAART) is the current treatment for controlling HIV viremia, and it is capable of reducing HIV production to undetectable levels. HAART provides indefinite viral suppression, enables immune function to be restored and dramatically decreases the mortality rate of HIV infected patients [1–3]. Nevertheless, the appearance of long-term side effects and emergence of viral resistance after prolonged use frequently weakens its effectiveness [4,5]. Various HIV-1 vaccine approaches have been developed, and tested in clinical trials, but none have yet proven effective [6,7]. Alternative therapies are therefore needed.

Gene therapy has evolved into a promising therapeutic modality to prevent the progression of HIV infection by introducing the anti-HIV transgenes into target cells to interfere with viral replication [8,9]. Recent vector improvements have enhanced their clinical use. Lentiviral vectors including HIV-1-derived vectors are promising tools for gene therapy applications because of their high transmissibility in both dividing and non-dividing cells and efficient long-term expression [10,11]. An ideal lentiviral-based system would be contained within a single vector, to avoid the need for high multiplicities of infection (MOI) multiple transductions of target cells, a process that would increase the risk of insertional mutagenesis [12].

With the development of safer lentiviral vectors, the current challenge for gene therapies is to efficiently regulate therapeutic gene expression. Amongst the existing inducible transcriptional gene regulatory systems, the Tet-regulatable system is the most widely used for controlling gene expression [13]. Tet-On transactivation system can be turned ‘on’ in the presence of the inducer doxycycline (Dox) and switched ‘off’ in its absence [14,15]. The Tet-On system has many important features for gene therapy because Dox has been broadly used as an antibiotic in humans and can be taken orally. In addition, the level of gene expression in individual cells correlates directly with the dose of inducer in dose-dependent manner [16], allowing a graded transcriptional response [17]. For these reasons, the Tet-On system could be applied to HIV-1 gene therapy.

Several engineered proteins have already been developed for HIV-1 gene therapy [18–21]. Antibody-based drugs are the largest class of protein therapeutics, as intrabodies. The intracellular expression of a single chain variable fragment (scFv) specifically recognizing matrix [22] or tat [23], HIV-1 proteins have been shown to efficiently inhibit HIV-1 replication in human T cells. However, the applications of therapeutic antibodies have several limitations regarding inadequate pharmacokinetics and ability to reach intracellular targets because the disulphide bonds required for structural stabilization do not form in the cytoplasm [24]. To overcome this shortcoming, protein scaffolds with the following attributes have been selected: high stability and solubility; high recognition affinity; and lack of disulphide bonds and/or glycosylation sites. Editing of the *CXCR4* gene in CD4^+^ T cells and *CCR5* gene in hematopoietic stem cells (HSC) using zinc-finger nuclease (ZFN) fusion proteins resulted in the expression of non-functional HIV co-receptors, and rendered these cells resistant to HIV-1 entry [25]. Ank^GAG^1D4, isolated artificial ankyrin protein, specifically recognizes capsid proteins, exhibited a significant antiviral effect, interfering with HIV-1 assembly [19,20].

Previously, we have designed a novel class of zinc finger proteins, 2-long terminal repeat zinc-finger protein (2LTRZFP), specifically designed to bind the conserved 2-long terminal repeat (2-LTR) circle junction of HIV-1 DNA. It showed high affinity for the integrase recognition sequence at the 2-LTR circle junctions and revealed the promising function of blocking viral integration into host chromosome at an early step of infection [26,27]. However, side or off-target effects of a constantly expressed transgene can occur in gene therapy applications [28].

In the present study, we have designed a next-generation, self-inactivating vector that contains the most recent features of the Tet-On system, allowing safe, efficient, and controllable intracellular expression of the 2LTRZFP protein. Here we evaluate its expression control and its antiviral activity in preventing viral DNA integration. In addition, we tested the controllable 2LTRZFP lentiviral vector in pluripotent stem cells and provide proof of concept for future clinical applications.

## Experimental

### Construction of the Dox-inducible lentiviral vectors

The pLVX-TetOne-Puro vector (Clonetech, Palo Alto, CA) was used as an acceptor for cloning the 2LTRZFP and Aart by using the fusion HD cloning system (Clonetech, Palo Alto, CA). Briefly, 2LTRZFP and Aart were amplified from CGW-*2LTRZFPmCherry* vector and CGW-*AartmCherry* vector, respectively. One microgram of genomic DNA was amplified by using Q5™ High-Fidelity DNA Polymerase (NEB Biolab, Ipswich, MA) with a pair of primers that matched 15-bp sequences at the ends of the linearized pLVX-TetOne-Puro acceptor vector. The PCR was performed under the following conditions: initial denaturation at 98°C for 30 s, followed by 35 cycles of denaturation at 98°C for 10 s, annealing at 50°C for 30 s, extension at 72°C for 30 s, and final extension at 72°C for 2 min. The PCR product was subsequently cloned into linearized pLVX-TetOne-Puro vector by the In-Fusion HD Cloning Kit (Clonetech, Palo Alto, CA) according to the procedure recommended by the manufacturer. The pLVX-TetOne-Puro vector carrying *2LTRZFPmCherry* or *AartmCherry* genes were named pLVX-TetOne-Puro-2LTRZFP or pLVX-TetOne-Puro-Aart, respectively.

### Production of lentiviral vectors

To produce vesicular stomatitis virus glycoprotein (VSV-G) pseudotyped lentivirus for induction of the gene of interest, HEK293T cells were co-transfected with 10 µg pLVX-TetOne-Puro vectors and packaging vectors including 10 µg psPAX2 and 5 µg pMD2.G vectors using TransIT-X2® Dynamic Delivery System (Mirus Bio, Madison, WI). The reagent–DNA complex was incubated with the cells for 72 h in a 37°C humidified incubator containing 5% CO_2_. The supernatants were harvested and filtered through 0.45-µm pore size filters (Millex-HA filter unit; Merck Millipore, Hessen, Germany). The viral vector titer was determined by transduction of 293T cells with serially diluted culture supernatants, treating with Dox for 3 days, and counting the number of mCherry-positive cells.

### Generation of the stable expressing SupT1

A total of 1 × 10^5^ SupT1 cells were mixed with culture supernatants containing Tet-On lentivirus harboring *2LTRZFPmCherry* or *AartmCherry* genes and spinoculated at 2000×***g*** at 32°C in the presence of 8 µg/ml polybrene (Sigma–Aldrich, St. Louis, MO) for 24 h. After incubation, the infected cells were washed three times with fresh growth medium and further cultured in freshly prepared RPMI medium containing 250 ng/ml puromycin and 10% FBS for 7 days. Puromycin-resistant clones were propagated for 7 days aliquoted and frozen with 10% DMSO in FBS. The SupT1 cell line transduced with Tet-On lentivirus vector carrying *2LTRZFPmCherry* and *AartmCherry* genes were named SupT1-Tet-On-2LTRZFP and SupT1-Tet-On-Aart, respectively.

### Optimization of Dox concentration for induction

A total of 1 × 10^5^ of SupT1-Tet-On-2LTRZFP or SupT1-Tet-On-Aart cells were cultured with various concentrations of Dox (Merck, Darmstadt, Germany) including 0, 0.1, 0.5, 1, 5, and 10 µg/ml in 24-well plate for 3 days. After incubation, the expression of mCherry fluorescence protein was captured by inverted fluorescence microscope and the transduction efficiency was determined by flow cytometer. An aliquot of this cell suspension was stained with 0.4% Trypan Blue dye and counted with an automated cell counter Countess® II (Thermo Scientific, Rockford, IL).

To test the stability of the transgene expression in Tet-On system, a total of 1 × 10^6^ wild-type SupT1, SupT1-Tet-On-2LTRZFP or SupT1-Tet-On-Aart cells were incubated with 1 µg/ml of Dox for 3 days. After incubation, all cells were washed with incomplete RPMI three times and fresh medium was added to continue culture. The percentage of mCherry expression was monitored by flow cytometry.

### Challenge with pseudotyped HIV-1

A total of 1 × 10^5^ SupT1-Tet-On-2LTRZFP or SupT1-Tet-On-Aart cells were plated in each 96-well plate containing 150 µl of complete RPMI medium with 1 µg/ml Dox for 3 days and then inoculated with VSV-G-pseudotyped HIV-1_NL4-3.Luc.R_^−^_.E_^−^ (50 ng of HIV-1 p24 per 10^5^ cells) through spinoculation at 2000×***g*** at 32°C for 1.5 h. The inoculated cells were cultured for 72 h and measured the luciferase activity by adding luciferase substrate according to the manufacturer’s procedure (Promega Corporation, Madison, WI).

### Determination of the antiviral activity against X4 tropic HIV-1 infection

The antiviral activity of the inducible 2LTRZFP expression was assessed by X4-tropic HIV-1_NL4-3_ infection. SupT1-Tet-On-2LTRZFP and SupT1-Tet-On-Aart cells were treated with 1 µg/ml of Dox for 3 days before HIV-1 challenge. A total of 1 × 10^6^ control SupT1 cells, SupT1-Tet-On-2LTRZFP and SupT1-Tet-On-Aart, one set induced by Dox and the other set not, were challenged with X4-tropic HIV-1_NL4-3_ at MOI of 10 for 16 h. Inoculated cells were washed three times with serum-free medium, and cultured in new growth medium. In addition, the SupT1-Tet-On-2LTRZFP and SupT1-Tet-On-Aart that first turned on gene expression were maintained in the medium in the presence of 1 µg/ml Dox throughout the experiment. All conditions were passaged (1:2) at 3-day intervals in order to maintain a cell density of approximately 10^6^ cells/ml. The level of HIV-1 replication was monitored by determining the p24 antigen in culture supernatants by ELISA (Bio-Rad, Marnes-la-Coquette, France). Cell pellets of all conditions were collected to measure the level of proviral integration via the *Alu-gag* qPCR assay as described previously [26].

### Expression of inducible 2LTRZFP in pluripotent stem cells

The non-integrating human induced pluripotent stem cell (hiPS) cell line (HFSK#4.hiPS) [29] and normal human ES cell (hESC) line (Chula2.hES) [30] were cultured in mTeSR1 medium (Stem Cell Technologies, Vancouver, Canada) for 3 days before transduction. The lentiviral vector was added to the cells at MOI of 20 with 8 µg/ml of polybrene (Sigma–Aldrich, St. Louis, MO) for 24 h. On the following days, the transduced cells were washed three times with mTeSR1 medium and further cultured with fresh mTeSR1 medium containing puromycin at 0.25 µg/ml for 5 days to select the transduced cells. Cells were cultured in mTeSR1 medium containing 1 µg/ml Dox for another 3 days to switch the transgene on prior to using the single cell selection method. The expression of 2LTRZFP corresponded with the mCherry expression detected by fluorescence microscopy.

### Statistical analysis

Results were represented as mean ± S.D. from three independent assays. The statistical comparisons were determined by one-way ANOVA with *P*-value considered as ***P<0.001; **P<0.01; *P<0.1; NS, not significant.

## Results

### Construction of a tetracycline-inducible lentiviral vector

**Figure 1:**
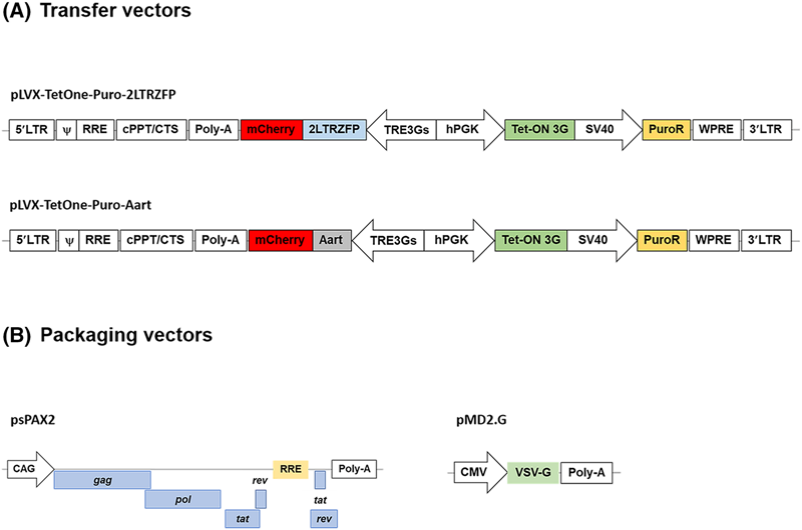
Schematic diagrams of Tet-On inducible lentiviral vectors and packaging vectors (**A**) The pLVX-TetOne-Puro vector served as the acceptor vector for cloning the full-length coding sequence of 2LTRZFP or Aart in BamHI or EcoRI recognition sites. (**B**) The packaging vectors for producing lentiviral particle. The psPAX2 plasmid encodes Gag, Pol, Rev, and tat and pMD2.G encodes the vesicular stomatitis virus G glycoprotein (VSVG) envelope protein.

A tetracycline (Tet)-inducible lentivirus system was designed to contain the most possible features to enable safe and efficient use. The pLVX-TetOne-Puro lentiviral vector enables transgene expression under the tight control of the TRE3G promoter that consists of seven repeats of a 19-bp tet operator sequence upstream of a minimal CMV promoter (Figure 1A). The Tet-on 3G transactivator is expressed in the reverse orientation and controlled by the phosphoglycerate kinase1 (PGK) promoter. This lentiviral vector also contains a puromycin resistance gene, allowing selection of stable clones (Figure 1A). To produce viral particles, recombinant vectors, pLVX-TetOne-Puro-*2LTRZFPmCherry* or pLVX-TetOne-Puro-*AartmCherry*, were co-transfected into HEK293T cells with plasmids psPAX2 and pMD2.G that produce the necessary packaging proteins, as outlined in Figure 1B.

### Optimization of Dox concentration for 2LTRZFP expression

**Figure 2:**
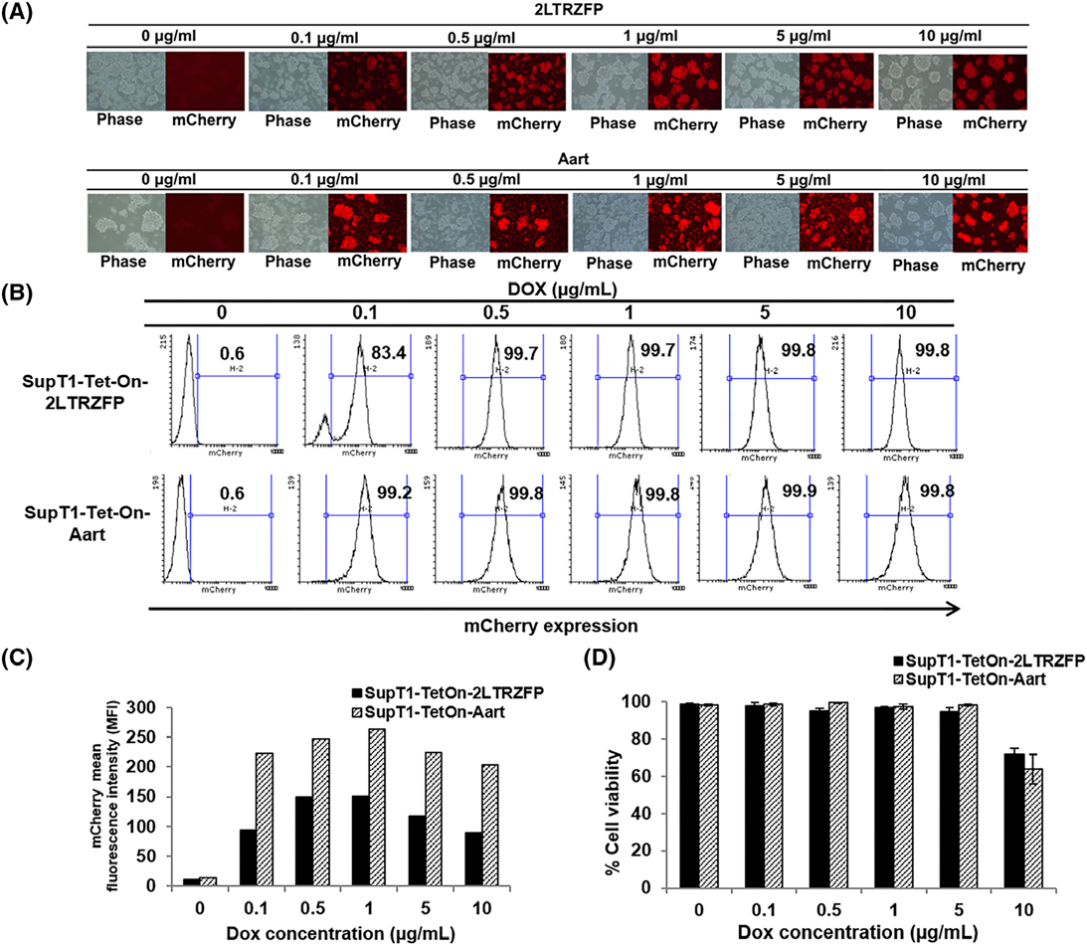
Dox optimization of 2LTRZFP expression in SupT1 cells (**A**) A puromycin-resistant SupT1 cell clone was incubated with a range of Dox concentrations for 72 h. The cell morphology and mCherry expression were observed by fluorescence microscopy. (**B**,**C**) The percentage and level of cells expressing mCherry were evaluated by flow cytometry. (**D**) Cell viability of transduced cells in all induction conditions was assessed by Trypan Blue exclusion using an automated cell counter Countess® II (Thermo Scientific, Rockford, IL). Results presented are from triplicate experiments (mean ± S.D.).

The recombinant lentiviral vectors carrying *2LTRZFPmCherry* or *AartmCherry* genes were transduced into SupT1 cell line. On the following day, the stable clones were selected by adding puromycin antibiotic (0.25 µg/ml). The survival cells in puromycin treatment were further propagated and used for optimization of Dox. Various concentrations of Dox were added to culture medium to activate transgene expression, *2LTRZFPmCherry* or *AartmCherry*. Expression of each was determined after 3 days of Dox induction, compared to without Dox. No expression of either transgene was observed in the absence of Dox. Expression was detected at 0.1–10 µg/ml Dox (Figure 2A). The expression level of *2LTRZFPmCherry* at all concentrations of Dox was lower than that of *AartmCherry* as determined by flow cytometry (Figure 2B,C). The induction efficiency of Dox at 0.1 µg/ml was lowest, 83.4% of the SupT1-Tet-On-2LTRZFP cells expressed detectable mCherry (Figure 2B). The mCherry expression was maximal at 0.5 and 1 µg/ml Dox (Figure 2B,C) with a low level of toxicity (Figure 2D). Higher levels of Dox, 5 and 10 µg/ml, did not further enhance mCherry expression (Figure 2C). In addition, the 10 µg/ml Dox reduced cell viability (Figure 2D). Taken together, the Tet-On lentiviral system efficiently introduced the therapeutic gene into target cells, controlled expression effectively in the absence of induction, and expressed efficiently with a low Dox concentration, 0.5–1 µg/ml.

### Stability of 2LTRZFP expression on induction by the Tet-On system

**Figure 3:**
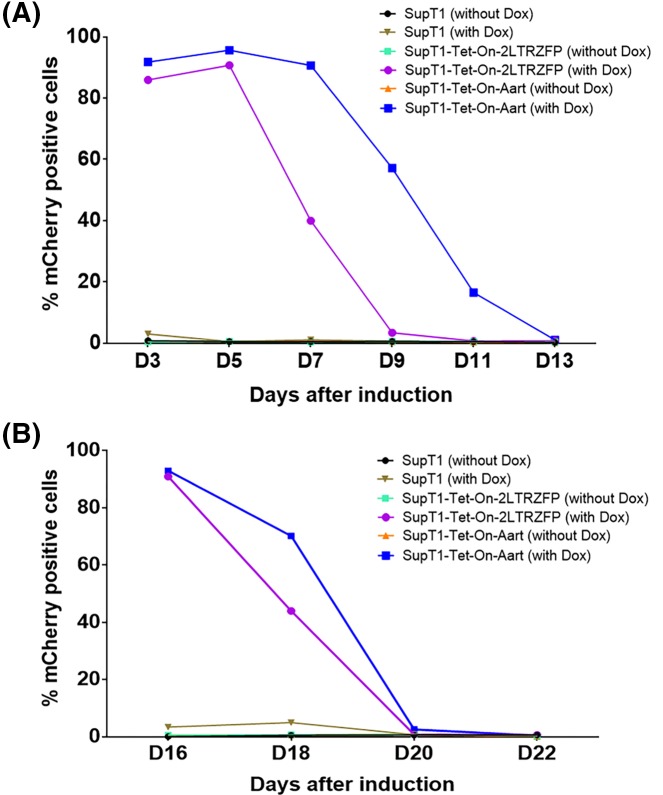
Stability of the inducible 2LTRZFP following sequential inductions with Dox The *2LTRZFPmCherry* expression was induced by the presence of Dox twice. SupT1-Tet-On-2LTRZFP cells were initially cultured with or without Dox for 3 days. After incubation, Dox was removed from the system to allow expression to decay. (**A**) After the first induction, expression of *2LTRZFPmCherry* was monitored for 13 days by flow cytometry. (**B**) After the mCherry expression diminished to the background level, the cells were again induced with Dox for 3 days and expression of mCherry was again monitored.

To assess the stability of 2LTRZFP expression, transgene expression in transduced SupT1 cells was monitored following the initial 3-day Dox treatment and its removal. The Tet-On system maintained 50% transgene expression in the SupT1-Tet-On-2LTRZFP and SupT1-Tet-On-Aart, for 6 and 9 days after Dox removal, respectively at the first induction (Figure 3A). After both sets of cells returned to baseline expression, they were again treated with Dox for 3 days. Transduced cells again expressed high levels of mCherry, but returned to baseline more quickly, by 4 days instead of 6 days for 2LTRZFP expression (Figure 3B). It is possible that the second induction did not reach maximal expression, due to incomplete recovery of transduced cells from the first induction.

### Inducible 2LTRZFP-mediated inhibition of pseudotyped HIV-1 infection

The protection of the transduced Sup-T1 cells from HIV-1 integration was evaluated by challenging these cells with a pseudotyped X4 tropic HIV-1 carrying a luciferase reporter gene (HIV-1_NL4-3.Luc.R_^−^_E_^−^/VSV-G). This virus is non-replicative chimeric virus which contains HIV-1 (strain NL4-3) core, the VSV-G envelope, and luciferase reporter gene. Infection by this virus requires that the reverse-transcribed copy of the virus genome integrates into the host chromosome. Once it does, it will also express luciferase indicating productive HIV-1 infection.

**Figure 4:**
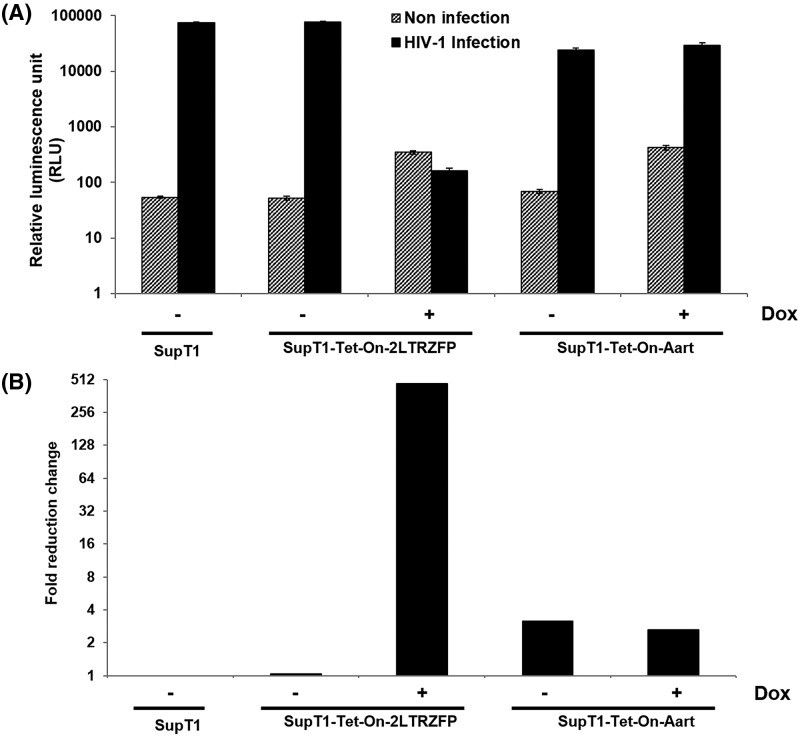
Inhibition of pseudotyped HIV-1 integration by inducible 2LTRZFP SupT1-Tet-On-2LTRZFP and SupT1-Tet-On-Aart were incubated in the presence or absence of Dox for 3 days after which all cells were challenged with X4-tropic NL4-3.Luc/VSV-G pseudotyped virus. (**A**) Luciferase activity was measured at day 3 post-infection. Results presented are from triplicate experiments (mean ± S.D.). (**B**) Data are shown as fold reduction in relative luminescence units (RLU) compared with the RLU of wild-type SupT-1 cells.

After the different SupT1 cell lines were induced with Dox for 3 days or not induced, they were challenged with the pseudotyped virus and HIV infection was quantitated by measuring luciferase activity. Only the SupT1-Tet-On-2LTRZFP cells exhibited inhibitory activity against HIV-1 infection as a result of Dox treatment (Figure 4A). Dox-treated SupT1-Tet-On-Aart did show a three-fold reduction in luciferase activity compared with non-transduced SupT1, but this level was similar without Dox treatment. However, the most dramatic decrease was 467-fold in Dox-induced SupT1-Tet-On-2LTRZFP (Figure 4B). This result demonstrates that the induced 2LTRZFP interfered with HIV-1 infection.

### Inducible 2LTRZFP-mediated inhibition of X4 tropic HIV-1 infection

**Figure 5:**
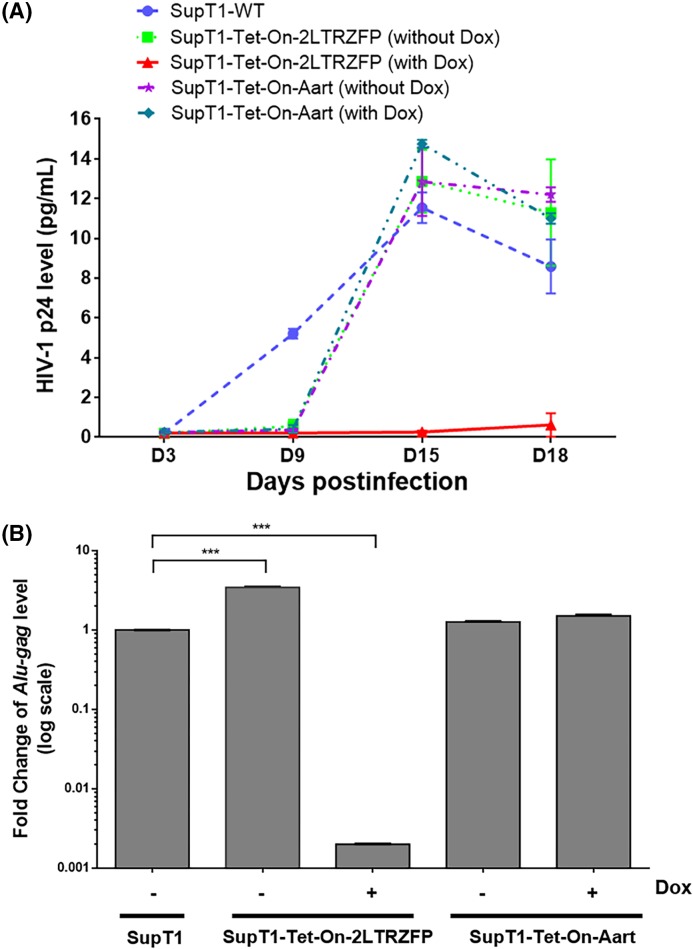
Kinetics of the antiviral activity of induced 2LTRZFP against replication of the HIV-1X4-tropic strain Wild-type SupT1, and SupT1-Tet-On-2LTRZFP and SupT1-Tet-On-Aart cell lines in the presence and absence of Dox, were challenged with HIV-1_NL4-3_ at an MOI of 10. (**A**) Every 3 days, supernatants and cells were collected and p24 protein measured by ELISA and (**B**) at day 12, viral integration was detected using *Alu-gag* real-time PCR. Relative *Alu-Gag* level was calculated compared with GAPDH (internal control) using the comparative *C*_T_ method (2^−ΔΔ*C*^_t_). Data are represented as fold change (log_10_) compared with the level of SupT1 control as mean ± S.D.; *n*=3; ***P<0.001.

**Figure 6:**
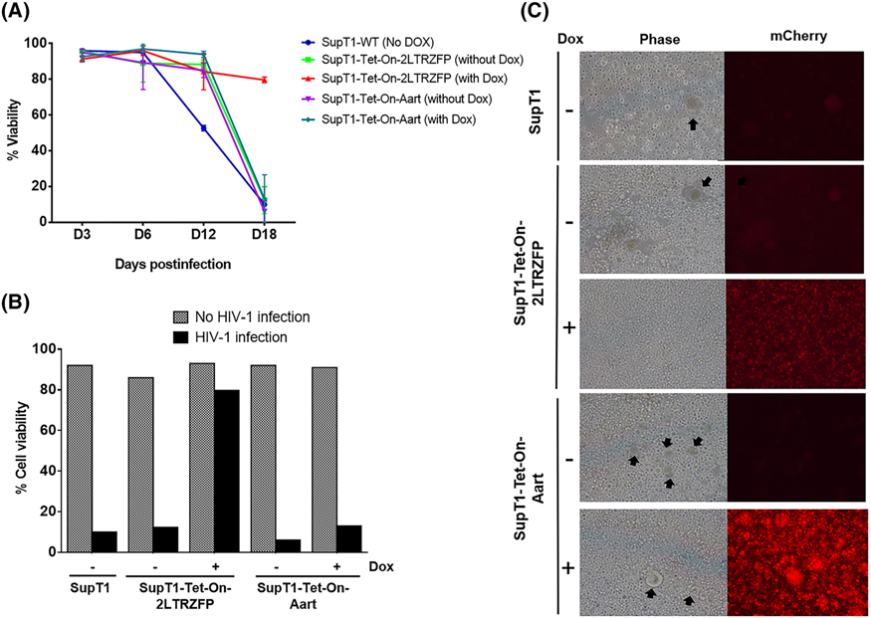
Cell viability and morphology of infected cells following HIV-1 challenge (**A**) Viability of Dox-induced or non-induced cells, inoculated with HIV-1 assessed throughout the experiment and (**B**) at D18 post-infection for viability by Trypan Blue exclusion. Results are from triplicate experiments (mean ± S.D.). (**C**) Cell morphology and mCherry expression were monitored by fluorescence microscopy at 100× magnification. Black arrows identify syncytia.

The antiviral activity of inducible 2LTRZFP was further assessed with wild-type HIV-1, strain NL4-3. Strain NL4-3 belongs to the T cell tropic X4 virus group which can infect and replicate in CD4-expressing cells that also express CXCR4. The supernatant collected from most cultures contained a high level of p24 protein at D15 post-infection that had begun to decrease by D18 due to HIV-1 induced cell death (Figure 5A). Only the Dox-induced SupT1-Tet-On-2LTRZFP cell line prevented p24 production through all 18 days post-inoculation. To confirm that the lack of p24 production was due to the lack of HIV-1 integration, the genomic DNA of the infected cells on D12 was extracted and the relative amount of provirus was measured by the *Alu-gag* qPCR method. The relative *Alu-gag* level was calculated relative to GAPDH (internal control) by the comparative *C*_T_ method (2^−ΔΔ*C*^_t_). The result revealed that the relative *Alu-gag* level of Dox-inducing SupT1-Tet-On-2LTRZFP was significantly lower than the SupT1 control (500-fold) and SupT1-Tet-On-2LTRZFP in the absence of Dox condition (1730-fold; Figure 5B). Of note, Dox-induced SupT1-Tet-On-2LTRZFP cell viability was maintained at almost 100% throughout 18 days post-inoculation (Figure 6A,B), without the cell–cell fusion detectable compared with other conditions (Figure 6C). This result confirmed that the induced 2LTRZFP significantly inhibited HIV-1 integration.

### Efficient control of 2LTRZFP expression in pluripotent stem cells

**Figure 7:**
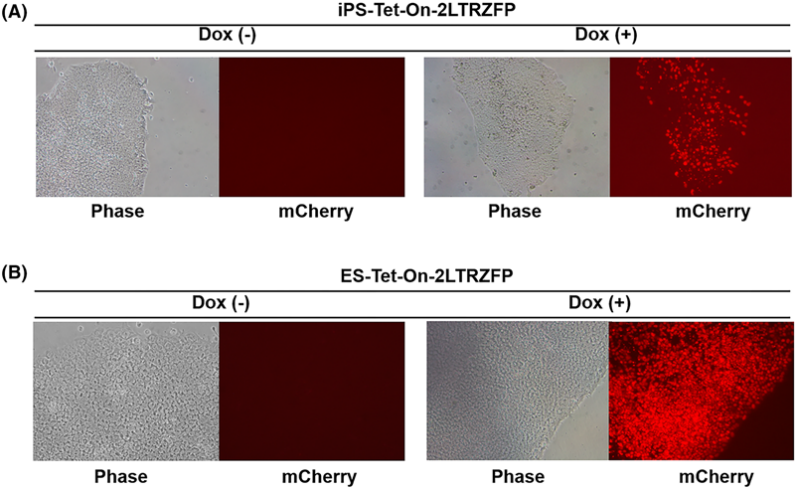
Expression of inducible 2LTRZFP in pluripotent stem cells (**A**) Human iPS cells and (**B**) ES cells were transduced with Tet-On-*2LTRZFPmCherry* lentiviral vector. After transduction, the transduced iPS and ES colonies were selected with puromycin for 5 days. The expression of mCherry was induced with 1 µg/ml Dox for 3 days and captured under fluorescence microscope at 100× magnification.

The Tet-On lentiviral vector was next used to introduce and express the *2LTRZFPmCherry* gene in pluripotent stem cells, i.e. human embryonic stem (ES) cells and induced pluripotent stem (iPS) cells. A small clump of wild-type pluripotent stem cells were transduced with the Tet-On lentivirus carrying the *2LTRZFPmCherry* gene. The transduced cells were selected with puromycin (0.25 µg/ml) for 5 days. Puromycin-resistant colonies were treated with 1 µg/ml of Dox and mCherry-expressing single clone were selected by the single cell dissociation method. The fact that *2LTRZFPmCherry* was expressed in the pluripotent stem cells indicates that the lentiviral vector combined with the Tet-On system offer a new method for delivering and integrating the *2LTRZFP* gene to pluripotent stem cells and expressing it at will by the addition of Dox (Figure 7).

## Discussion

The ability to regulate expression of a protective or therapeutic gene would be ideal for situations in which the level of its protein product may need to be controlled. Situations in which the protein is required periodically or if overexpression causes adverse side effects [13]. The Tet-On version of the system used here activates transcription in the presence of physiologically relevant doses of Dox. In the present study, the ‘all-in-one’ Tet-One vector encodes the Tet-On 3G transactivator protein which specifically binds to the *P*_TRE3G_ inducible promoter in the presence of Dox and consequently activates transcription of the downstream gene of interest, *2LTRZFP*. The expression of 2LTRZFP was induced with a low concentration of Dox, in the range utilized in clinical practice [31]. In contrast, no 2LTRZFP expression is observed in the absence of Dox, indicating that the *P*_TRE3G_ inducible promoter is tightly controlled, allowing very low if any basal expression, but high level expression upon activation.

Dose–response optimization is crucial both to identify the minimal level needed for therapeutic gene activation and to avoid toxicity and maintain viability. This system responded to Dox over a broad concentration range (0.1–10 µg/ml. However, SupT-1 treated with the highest concentration of Dox did display reduced cell viability indicating that 10 µg/ml is beyond the therapeutic range (Figure 2).

To assess the stability of the antiviral protein, 2LTRZFP, in this induction system, its expression was switched on/off two times during 22 days in culture. Following the first Dox induction and withdrawal, maximal expression was maintained for 2 days and fell to 50% cell expression by 4 days. Following the second induction, the number of cells expressing dropped to 50% by the next day. It is possible that the second induction did not cause maximal expression, perhaps due to incomplete recovery of the transduced cells after the first induction. The maintenance of stable expression and their ability to express when needed are both important attributes. Long-term activation of a transgene may need to be considered to avoid adverse side effect(s) [13].

Most importantly, the inducible 2LTRZFP exhibited high efficiency protection from HIV-1 infection. In case of pseudotyped NL4-3 infection, 2LTRZFP inhibited HIV-1 infection 460-fold in comparison with non-transduced and non-Dox induced cells. 2LTRZFP also inhibited viral integration and therefore infection of wild-type NL4-3 as evaluated by *Alu-gag* qPCR and p24 antigen release, respectively. Furthermore, 2LTRZFP also protected SupT1 cells from forming syncytia that lead to cell death. This inducible expression system for 2LTRZFP provides a promising approach for regulated HIV gene therapy.

HIV stem cell gene therapy is a promising strategy for life-long protection of HIV-1 infected individuals. However, there are some roadblocks to this approach. For instance, off-target and adverse side effects of transgene expression remain issues [32]. However, 2LTRZFP acts as a steric barrier between HIV-1 integrase and its substrate, which specifically targets an 18-bp region of the 2-LTR-circle junction. This site is highly conserved amongst HIV-1 strains. The conserved CA and TG dinucleotides at the end of the viral DNA precursor are vital to maintain an integrative element [33]. Mutation, insertion or deletion at the circle junction sequence results in defective integration into the host genome [34]. In addition, latent HIV-1 reservoirs remain a major obstacle for all HIV therapies. However, a dCas9-synergistic activation mediator (dCas9-SAM) system has been used to reactivate HIV-1 in cell lines [35,36]. Before reactivation, it would be useful to introduce 2LTRZFP into the patients’ HSC to protect them from further HIV-1 spread and to minimize or prevent additional latent HIV-1 reservoirs from developing.

Expression of transgenes in pluripotent stem cells could face the possibility of transgene silencing in a promoter-dependent manner or down-regulation of transgenes upon cell differentiation. However, several studies have successfully expressed transgenes in pluripotent stem cells [37–39]. The Sleeping Beauty (SB) transposon system is a powerful tool for introducing genetic information for stable expression of CD4ζ in hES and iPS cells and their differentiated NK cells *in vivo* and *in vitro* [39]. Using the Tet-On drug-inducible gene expression system with the PiggyBac transposon has achieved activation of transgene expression in human pluripotent stem cells [40]. Moreover, using the Tet-On inducible PiggyBac system has been reported to overcome the silencing of transgene expression during human pluripotent stem cells differentiation [41–43].

Regulatable systems have also been developed for gene therapy as well as in pluripotent stem cells. The Tet-regulatory system has been applied with CRISPR/Cas9 genome editing to regulate the vascular endothelial growth factor receptor 2 (VEGFR2/*Flk1*) gene in pluripotent stem cells [44]. TALEN-mediated targetting of the AAVS1 site has also been used in human pluripotent stem cells, controlled by a Tet-regulatory system [45].

Lentiviral vectors have been engineered using Tet-regulatory systems for *in vivo* delivery to the central nervous system [46] and *ex vivo* in a rat model for Huntington disease [47]. Recombinant adeno-associated virus – Tet regulatory system has also been demonstrated in the rat to regulate the Glial cell line-derived neurotrophic factor (GDNF) gene expression for treating Parkinson’s disease [48]. Tet-dependent regulatable gene expression provides many advantages over other regulatory gene expression systems. Because the inducer, Dox, has been used as antibiotic for several decades, it is known to have no toxicity at optimal dose for activation, has high clearance rate from body, and could be a drug of choice for long-term expression of desired transgenes [13].

Remarkably, the Dox-inducible 2LTRZFP scaffold could be expressed in pluripotent stem cells, both iPS and ES cells. Its expression was stable during passage, when Dox was present. The TRE3G transactivator was key to activate transgene expression and functioned in pluripotent cells [40]. As further proof of concept, these genetically modified pluripotent stem cells will be further characterized, for the stability of transgene expression, differentiation toward hematopoietic progenitors and anti-HIV-1 activity *in vitro*. The information obtained in these *in vitro* studies could pave the way for testing the safety and antiviral performance *in vivo* with humanized mouse and non-human primate models.

## Abbreviations

Dox: doxycycline
ES: embryonic stem
HAART: highly active antiretroviral therapy
HSC: hematopoietic stem cell
iPS: induced pluripotent stem
MOI: multiplicity of infection
Tet: tetracycline
VSV-G: vesicular stomatitis virus glycoprotein
2LTRZFP: 2-long terminal repeat zinc-finger protein
